# Aberrant Immune Responses in a Mouse with Behavioral Disorders

**DOI:** 10.1371/journal.pone.0020912

**Published:** 2011-07-20

**Authors:** Yong Heo, Yubin Zhang, Donghong Gao, Veronica M. Miller, David A. Lawrence

**Affiliations:** 1 College of Natural Sciences, Catholic University of Daegu, Kyongsan-si, Republic of Korea; 2 Wadsworth Center, New York State Department of Health, Albany, New York, United States of America; 3 University at Albany School of Public Health, Albany, New York, United States of America; Cardiff University, United Kingdom

## Abstract

BTBR *T+tf*/J (BTBR) mice have recently been reported to have behaviors that resemble those of autistic individuals, in that this strain has impairments in social interactions and a restricted repetitive and stereotyped pattern of behaviors. Since immune responses, including autoimmune responses, are known to affect behavior, and individuals with autism have aberrant immune activities, we evaluated the immune system of BTBR mice, and compared their immunity and degree of neuroinflammation with that of C57BL/6 (B6) mice, a highly social control strain, and with F1 offspring. Mice were assessed at postnatal day (pnd) 21 and after behavioral analysis at pnd70. BTBR mice had significantly higher amounts of serum IgG and IgE, of IgG anti-brain antibodies (Abs), and of IgG and IgE deposited in the brain, elevated expression of cytokines, especially IL-33 IL-18, and IL-1β in the brain, and an increased proportion of MHC class II-expressing microglia compared to B6 mice. The F1 mice had intermediate levels of Abs and cytokines as well as social activity. The high Ab levels of BTBR mice are in agreement with their increased numbers of CD40^hi^/I-A^hi^ B cells and IgG-secreting B cells. Upon immunization with KLH, the BTBR mice produced 2–3 times more anti-KLH Abs than B6 mice. In contrast to humoral immunity, BTBR mice are significantly more susceptible to listeriosis than B6 or BALB/c mice. The Th2-like immune profile of the BTBR mice and their constitutive neuroinflammation suggests that an autoimmune profile is implicated in their aberrant behaviors, as has been suggested for some humans with autism.

## Introduction

Development of an appropriate experimental animal model is critical if we are to extend our knowledge of the underlying causes of abnormal neurobehaviors. Numerous bidirectional immune and nervous system interactions are known, but we lack an understanding of the immunological mechanisms implicated in neuroendocrine developmental effects such as the behavioral disorders of autism. Aberrant immunity has been reported to be associated with autism and autism spectrum disorders (ASDs); however, there have been no studies mechanistically connecting a particular immune activity with the aberrant behaviors of individuals with an ASD [1]–[4]. For example, serum IgE levels have been reported to be normal [5], [6] or elevated [7], [8], and mastocytosis has been reported to be associated with ASD [9]. There does seem to be consistency regarding elevated levels of plasma cytokines and neuroinflammation (elevated levels of cytokines in the brain) being associated with ASD [10]–[14].

Among the inbred mouse strains that have been tested for abnormal behaviors, BTBR*T*+*tf*/J (BTBR) mice were among the most autism-like strains; BTBR mice display both low social approach and resistance to change in routine with the water maze assay, which is a behavior consistent with an autism-like phenotype [15]. BTBR mice also show low reciprocal social transference for food, high levels of repetitive self-grooming, low levels of social approach and juvenile play, and an unusual pattern of ultrasonic vocalizations; these traits respectively reiterate the impaired communication, repetitive behavior, and lowered reciprocal social interactions of autistic humans [15]–[18]. BTBR mice have severely reduced corpus callosum and hippocampal commisures [19], and reduction of corpus callosum and hippocampal commisures has been observed in some autistic children [20], [21]. Results on neuroanatomical and behavioral evaluations of BTBR mice are being accumulated, but no reports are available on the immunological characteristics of these mice. If cross-talk between the immune system and the nervous system [22] and the putative contribution of autoantibody (AutoAb)-mediated neuroinflammation to pathogenesis of ASDs are considered together, a systemic assessment of immune functions in BTBR mice could provide important clues to help to delineate the involvement of the immune system in the incidence and progression of ASD.

Immune system activities have been implicated in the development of ASD as well as in some of the associated pathophysiology. A broad spectrum of immune abnormalities, including aberrant mucosal immunity, has been reported for autistic subjects [23]. Additionally, serum Abs against central nervous system (CNS) antigens (Ags), and maternal Abs to fetal brain proteins, have been associated with ASD [23]–[26]. Maternal IgG reactive to fetal brain proteins contributing toward ASD development has been experimentally shown via induction of behavioral alterations in offspring mice or monkeys that were prenatally exposed to serum IgG obtained from mothers of autistic children [27], [28]. Thus, neuroinflammatory processes in the brain may play a role in the induction of the autistic behavioral changes induced by Abs.

To determine the relationship between the immunoreactivity and the abnormal behaviors that have been observed in BTBR mice, we analyzed various immune aspects of these mice, including presence of Abs to brain Ags, immune cell distribution in the periphery and brain, expression of proinflammatory cytokines in the brain, Ab production to KLH and host resistant against *Listeria monocytogenes* (LM, an intracellular pathogen). Not surprisingly, analyses of F1 offspring from two different crosses using the BTBR strain have indicated that the offspring have less anti-brain activity and neuroinflammation than is seen for the homozygous BTBR strain, but offspring from BTBR dams have greater anti-brain responses than offspring from B6 dams. The BTBR strain has elevated B cell activity that appears to have a predominant Th2-like profile, which may account for the BTBR's elevated immunity to brain Ags. Although the maternal environment has an influence on the immune status of the offspring, the BTBR genetic influence on behavior is suggested since the F1 mice of BTBR and B6 matings appear to have normal behavior although the F1 mice have less sociability than B6 mice.

## Results

### Enhanced amount of IgG deposited in the brain of BTBR mice

**Figure 1:**
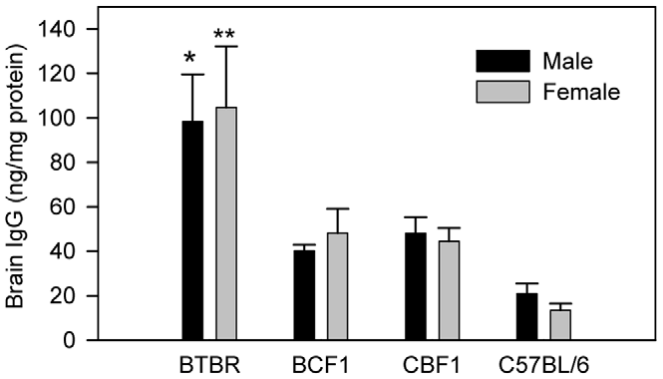
BTBR mice have an enhanced amount of IgG in their perfused brains. Whole brain homogenates were obtained from 4 BTBR males or females, 3 BCF1 males or females, 4 CBF1 males or females, and 5 B6 males or 4 females at postnatal day 21. Mice were perfused with PBS prior to the brain collection. *, *p*<0.05 *vs.* B6 males; **, *p*<0.01 *vs.* B6 females.

One of the major characteristics of autoimmune neurologic disorders is the generation of AutoAbs with specificity for various components of the nervous system [22], [29]. Since various AutoAbs against central nervous tissue antigens have been reported from autistic subjects [30], we queried whether a similar phenomenon was observable in BTBR mice. Human studies performed for evaluation of Abs against brain antigens have used serum as a source of Abs and specific neuronal proteins or else human brain protein medleys as sources of brain antigens [24], [25], [28], [31]–[35]. In our study, we first measured the amounts of IgG deposited in the whole brain or in separate brain regions of perfused mice; the presence of IgG was determined by ELISA, and was compared among the BTBR and B6 strains and their offspring. B6 mice were chosen as a control strain since, unlike BTBR mice, B6 mice demonstrate normal social behaviors and B6 mice have previously been used for the comparative behavioral analyses [15], [19], [36]; additionally, B6 mice are considered immunologically normal, and they have the same major histocompatibility complex (H2^b^) as BTBR mice. The IgG levels in whole-brain homogenates of BTBR mice (male: 98.4±21.3, female: 104.8±27.4 ng/mg protein) were approximately 2-fold higher than those of the F_1_ offspring (BCF1 male: 40.2±2.8, BCF1 female: 48.2±11.0, CBF1 male: 48.0±7.4, CBF1 female: 44.5±5.9 ng/mg protein), and 4-fold higher than those of the B6 control mice (male: 21.1±4.4, female: 13.6±3.0 ng/mg protein) (Fig. 1). There was no sexual dimorphism, but there was a significant strain difference (F = 11.45, DF = (3, 20), p<0.001); *post-hoc* analysis indicates BTBR brains contained significantly (p<0.001) more IgG than did B6 brains, but there were no differences between the F_1_ offspring and between F1 offspring and B6 mice.

Although there was no gender difference within a strain with regard to IgG deposited in the brains (Fig. 1), we still separately evaluated males and females for the regions with deposited IgG. Again, there were no significant gender differences; therefore, the results of males and females of each strain were pooled (Table 1). Based on two-way ANOVA, the only brain regions that significantly differed were substantia nigra (SN) and hippocampus (HC); additionally, regional amounts of IgG in BTBR mice differed from that of all of the other strains, and BCF1 results differed from those of B6. Overall, the SN was the region that showed the greatest accumulation of deposited IgG, and the striatum (STR) or HC had the lowest amounts of IgG.. The increased amount of IgG in the brains of the BTBR mice compared to those of B6 mice could not be further delineated by immunohistochemistry, in that perfused non-fixed brain sections from BTBR and B6 mice that were washed in vitro with PBS demonstrated no obvious differences in IgG distribution (data not shown).

**Table 1:** Levels of IgG present in brain regions.^a^

IgG level (ng/mg protein)^b^						
Strain	Cortex^c^	Striatum^d^	Hippocampus^e^	Hypothalamus^f^	Substantia nigra^e^	Cerebellum^d^
BTBR	151±41	103±26	94±17	110±24	211±35	159±36
BCF1	62±9	66±12	58±5	76±12	109±25	74±10
CBF1	42±2	40±4	35±4	54±3	56±3	59±11
B6	23±7	20±7	22±6	23±4	27±7	29±8

^*a*^Each brain region was isolated from perfused postnatal day 21 mice, and the homogenates were used for evaluation of IgG level deposited in each region. Number of mice was 8 for BTBR, and 6 for the other strains. The results are expressed as mean ± SEM. The IgG level in each brain region was significantly different among the strains (*p*<0.05).

^*b*^IgG levels in brain region of the mouse strains were significantly different by two-way ANOVA with BTBR vs. other strains and BCF1 vs. B6; SN also differed from HP.

^*c*^ *post-hoc* test indicates that the regional IgG level of BTBR differs from that of B6 and CBF1.

^*d*^ *post-hoc* test indicates that the regional IgG level of BTBR differs from that of B6.

^*e*^ *post-hoc* test indicates that the regional IgG level of BTBR differs from that of B6 and CBF1, and BCF1 differs from that of B6.

^*f*^ *post-hoc* test indicates that the regional IgG level of BTBR differs from that of B6, and BCF1 also differs from B6.

### Social behavior

**Figure 2:**
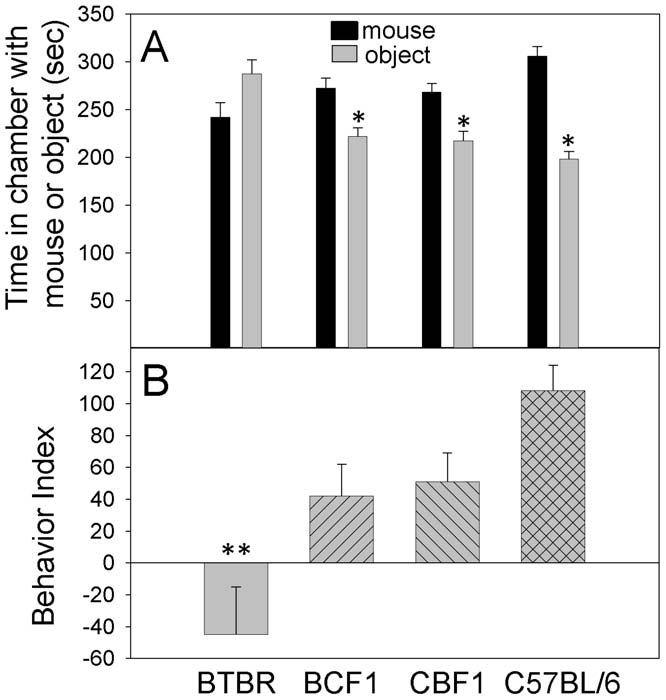
BTBR mice lack sociability. After a 10 min period for the test mouse to become acquainted with the three chamber box, the test mouse was assessed (10 min) for the amount of time spent in the side chamber with the caged novel (BALB/cByJ) mouse versus the opposite side chamber with an empty cage. C57BL/6 (B6), CBF1, and BCF1 mice spent significantly (*) more time in the chamber with the novel mouse (A); however, based on the behavior index (time with mouse minus time with object), the behavior of the BTBR mice was significantly different (**) from that of the CBF1 or B6 mice (B).

Based on the time spent in the chamber with the novel mouse *vs.* the time in the chamber with the empty cage, adult (≥pnd70) BTBR mice did have less sociability than the B6, CBF1, and BCF1 strains (Fig. 2). This is consistent with the previous evaluation of BTBR and B6 mice by this behavior assay [15], [17]. There were no significant differences for gender. Although unlike the BTBR mice, the F1 strains spent more time with the novel mouse than with the novel object (Fig. 2A), they did display less sociability than the B6 mice (Fig. 2B). Thus, the intermediate behaviors of the F1 mice appear to reflect the intermediate amounts of IgG in the brains of the F1 mice (Fig. 1). Kruskal-Wallis one-way ANOVA indicated significant strain differences, and Dunn's pairwise analysis indicated that BTBR mice had less sociability than the CBF1 and B6 mice.

### Upregulation of IgG and brain-reactive IgG expression in the peripheral blood of BTBR mice

**Figure 3:**
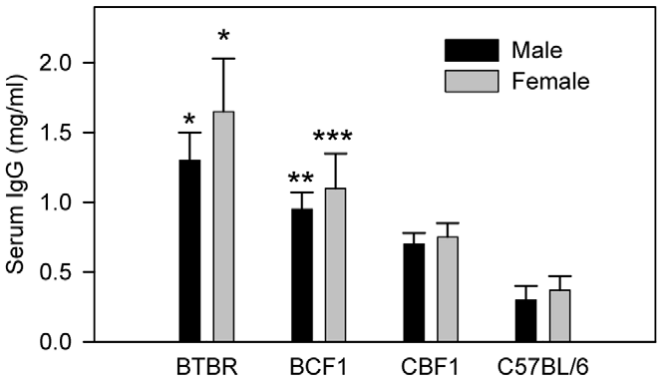
BTBR and BCF1 mice have significantly higher serum IgG levels than those of CBF1 or C57BL/6 (B6) mice. Sera were obtained from postnatal day 21 mice. The numbers of mice were 14 for BTBR male, 17 for BTBR female, 9 each for BCF1 and CBF1 male or female mice, and 11 for each B6 male or female. *, *p*<0.001 *vs.* CBF1 and B6 males or females; **, *p*<0.05 vs. B6 males; and ***, p<0.01 *vs.* B6 females.

**Figure 4:**
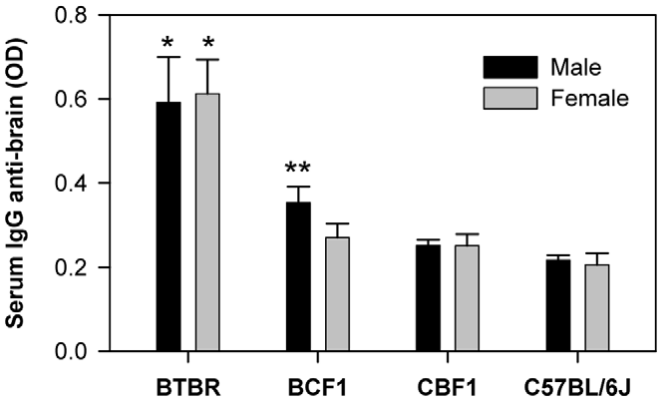
BTBR and BCF1 mice have significantly higher amounts of serum anti-brain antibodies than CBF1 or C57BL/6J (B6) mice. Sera from postnatal day 21 mice were added to wells coated with BALB/c SCID whole brain proteins (10 µg/well). The numbers of sera were 6 for BTBR male or female, 6 for BCF1 male, 3 for BCF1 female, 5 for CBF1 male or female, and 7 for B6 male or female. The level of brain-reactive IgG was determined by using the HRP-conjugated goat anti-mouse IgG. *, *p*<0.01 *vs.* B6 males or females, and **, *p*<0.05 *vs.* B6 males.

Since there was significantly more IgG present in the perfused brains of BTBR mice than in those of the F1 offspring or B6 mice, we measured the amounts of total serum IgG (Fig. 3) and the levels of serum IgG binding to brain homogenates (Fig. 4) of pnd21 BTBR, BCF1, CBF1, and B6 mice. The level of total IgG was highest in BTBR mice, lowest in B6 mice, and intermediate in BCF1 and CBF1 mice. Two-way ANOVA indicated no interaction or gender significance, but there was strain significance (F = 20.91, DF = (3, 80), p<0.0001). One-way ANOVA (Kruskal-Wallis test) based on strain indicated p<0.0001 and Dunn's *post-hoc* test indicated BTBR mice had higher total IgG levels (p<0.001) than did CBF1 and B6 mice, and BCF1 mice had higher total IgG levels (p<0.001) than did B6 mice. Based on a comparative study of B6 mice with five other strains, the IgG levels of BTBR or BCF1 pups are not higher than those of B6 pups because of abnormally low IgG levels in B6 mice since B6 mice have relatively high levels of serum IgG (http://phenome.jax.org) and of peripheral blood B cells [37].

The presence and amounts of brain-reactive IgG in serum samples from BTBR mice were compared with those assessed for BCF1, CBF1, and B6 mice. Homogenates from whole brain or from each of six different brain regions from SCID mice were used as Ags to detect brain-reactive IgG through ELISA. As for total serum IgG levels, the anti-brain Ab levels in sera were not different by gender, but they were significantly different based on strain (F = 20.99, DF = (3, 37), p<0.0001). The level of brain-reactive IgG against whole brain homogenates was higher in BTBR mice than in CBF1 or B6 mice (p<0.001), and male BCF1 anti-brain levels were greater (p<0.05) than those of B6 mice (Fig. 4). Analysis (three-way ANOVA for sex, strain and brain region) of serum IgG binding to various brain regions (cortex, CTX; STR; HC; hypothalamus, HT; SN; cerebellum, CB) showed overall significance for strains (F = 118.502, DF = 3, p<0.001) that the brain-reactive IgG levels were highest in the brain regions tested with sera from BTBR mice; intermediate binding activity was obtained with sera from BCF1 mice, and sera from B6 mice showed the lowest binding; there were no differences between CBF1 and B6 mice (Table 2). Additionally, there were differences in the amount of IgG binding to SN *vs.* all other regions and HT *vs.* HC and CB. As shown for whole brain homogenate (Fig. 4), there were no significant differences between sexes within a strain; however, BCF1 males exhibited significantly higher amounts of brain-reactive IgG against CTX, SN, and CB homogenates than did B6 males, and no corresponding finding was observed for the females of these two strains. These observations suggest that the antigenic targets of the elevated levels of the IgG to brain Ags are not focused in any specific brain regions, but instead are reactive to Ag(s) common throughout the brain regions; however, the regional differences may relate to the proportion of different cell types in each region or relative accessibility of serum constituents.

**Table 2:** Serum IgG binding to Ags of various brain regions.^a^

		Level of brain-reactive IgG (optical density)^b^					
Strain	Sex	Cortex (CTX)	Striatum (STR)	Hippocampus (HC)	Hypothalamus (HT)^c^	Substantia nigra (SN)^d^	Cerebellum (CB)
BTBR	Male	0.50±0.06	0.49±0.04	0.57±0.06	0.44±0.03	0.42±0.03	0.44±0.03
	Female	0.56±0.05	0.56±0.05	0.62±0.05	0.51±0.05	0.48±0.05	0.47±0.05
BCF1	Male	0.38±0.04	0.39±0.04	0.40±0.04	0.36±0.03	0.33±0.03	0.34±0.03
	Female	0.31±0.03	0.32±0.02	0.34±0.02	0.30±0.02	0.28±0.01	0.29±0.02
CBF1	Male	0.27±0.02	0.29±0.03	0.31±0.03	0.27±0.03	0.23±0.02	0.24±0.02
	Female	0.28±0.02	0.29±0.03	0.32±0.02	0.28±0.02	0.24±0.01	0.26±0.02
B6	Male	0.24±0.02	0.28±0.04	0.31±0.04	0.26±0.04	0.19±0.02	0.20±0.01
	Female	0.25±0.03	0.28±0.05	0.29±0.05	0.25±0.05	0.21±0.03	0.22±0.03

^*a*^Sera from postnatal day 21 mice were added to wells coated with each brain region protein (10 µg/well). Optical densities were measured as described in Figure 2. The results are expressed as mean ± SEM.

^*b*^Significant differences were assayed by three-way ANOVA (sex, strain, region) followed by *post hoc* tests. There were no sex differences within a strain. Differences (p value) were as follows: BTBR *vs.* C57BL/6J (B6) (0.009), CBF1 (0.01), and BCF1 (0.013); BCF1 *vs* B6 (0.017) and CBF1 (0.025).

^*c*^Significant difference in HT *vs.* CB (0.005) and HC (0.006).

^*d*^Significant difference in SN *vs.* CTX (0.004), STR (0.004), HC (0.004 ), HT (0.005), and CB (0.003).

When all of our IgG results are considered together, a consistent upregulation of the levels of IgG was apparent in the sera and brains of BTBR mice, relative to what was seen for the other strains; also, BCF1 offspring (BTBR dams and B6 sires), especially males, showed higher IgG levels than did B6 mice. However, the IgG levels did not differ between CBF1 and B6 mice. A high constitutive polyclonal B cell activation in the BTBR mice could lead to high endogenous systemic inflammation.

### Blood brain barrier (BBB) integrity

**Figure 5:**
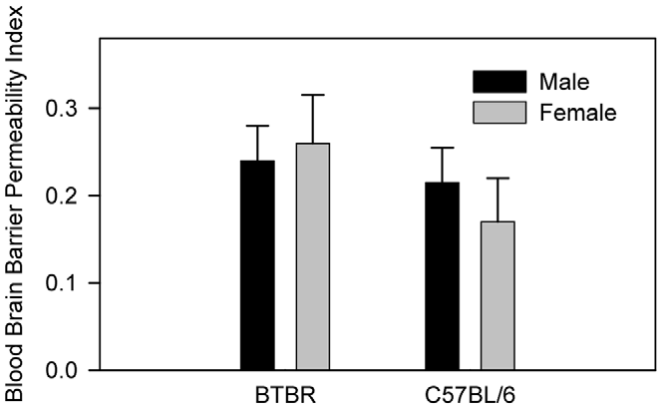
No difference in BBB permeability in BTBR mice relative to B6 mice. Evans blue solution (50 µg/g body weight) was injected into the peritoneum; 5 h later, peripheral blood was obtained through cardiac puncture. Brains from the perfused mice were incubated in formamide solution for 72 h for extraction of the dye, and the supernatants were collected through centrifugation. Optical density of plasma or brain supernatant sample was measured at 620 nm. No statistical significance was observed between BTBR and C57BL/6J mice. Brains were removed after PBS perfusion and weighed.

BBB leakage or increased BBB permeability can cause extravasation of virus, immunoglobulin, or toxic molecules into brain tissues [38]–[40]. To investigate whether increased BBB permeability was a factor contributing to the increased amount of deposited IgG in the brain of the BTBR mice, we assessed leakage of Evans blue (EB) dye into the brain after intraperitoneal injection of the dye (Fig. 5). The BBB permeability index, which reflects the amount of EB dye per unit weight of brain relative to the amount of dye per unit volume of plasma, did not differ between BTBR and B6 mice.

### Immune cell subpopulations in the peripheral organs and brain of BTBR mice

**Figure 6:**
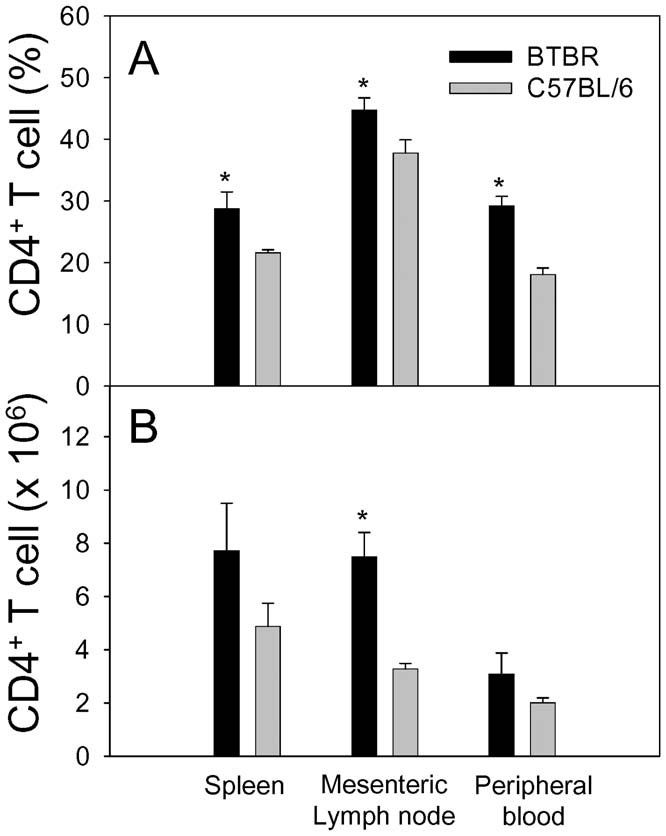
Elevated number of CD4^+^ T cells in various peripheral organs of BTBR mice, compared with those of B6 mice. Spleens, mesenteric lymph nodes, and peripheral blood were obtained from postnatal day 21 male or female mice (n = 3 for each sex per strain). Since there were no gender differences within a strain, the results were pooled by strain. Percentages (A) or absolute numbers (B) of CD4^+^ T cells among CD45^+^ cells were determined by flow cytometric analysis. Number of cells indicates the number per spleen, three pooled lymph nodes, or 1 ml of blood. *, BTBR *vs.* C57BL/6 (*p*<0.05).

The various types and proportions of immune cells distributed throughout the peripheral immune organs and brain have not been previously evaluated for BTBR mice. Since some immune cell types in the periphery or CNS, such as microglial cells or CD4^+^ T cells, have been suggested to be associated with pathogenesis of neuroinflammatory disorders, including autism [23], [41]–[44], we evaluated proportions and actual numbers of major immune cell types in the spleen, mesenteric lymph nodes, peripheral blood, and brain of BTBR mice, and compared the levels with those for B6 mice (Tables 3 & 4, Fig. 6). Given our described result showing relatively high levels of IgG in BTBR mice, we also enumerated plasma cells. The number of CD8^+^ T cells was significantly greater in the mesenteric lymph nodes of the BTBR mice than those of B6 mice, but this difference was not obtained for analysis of spleens or blood (Table 3). Although the serum IgG levels were elevated in BTBR mice compared with the IgG levels of B6 mice, the pnd21 BTBR mice did not have any significant differences from B6 mice with regard to the number of B cells or plasma cells in the spleen, lymph node or blood (Table 3). BTBR and B6 mice (pnd60) also were assayed for splenic B cell numbers and the levels were still equivalent (BTBR, 38.3±2.6 and B6, 37.4±2.7×10^6^/spleen). A subset of mice were assayed for B1 (CD19^+^/CD5^+^) cells since B1 cells are often implicated in autoimmune processes. The percentages of splenic or blood B1 cells were not different between BTBR and B6 mice (2.9±0.8% *vs.* 3.2±0.7% for spleen and 0.4±0.2% vs. 0.5±0.2% for blood). The percentage of plasma cells (CD45^+^/CD138^+^) cells in the bone marrow also was not significantly higher in the BTBR mice (BTBR, 4.2±0.6 and B6, 2.9±0.4%). The BTBR mice did have a greater number of CD4^+^ T cells in the mesenteric lymph nodes (Fig. 6). CD4^+^ T cell differences in both the proportions in peripheral blood (BTBR male: 30.0±1.8, BTBR female: 28.4±3.0, B6 male: 17.0±1.9, B6 female: 19.1±1.0%) and the actual numbers in mesenteric lymph nodes (BTBR male: 8.2±1.6, BTBR female: 6.8±1.0, B6 male: 3.5±0.2, B6 female: 3.2±0.3×10^6^ cells) attained statistical significance.

**Table 3:** Distribution of lymphoid cell subpopulations in the peripheral immune organs.

Organ	Strain	CD8^+^ T cell	CD19^+^ B cell	CD138^+^ plasma cell
Percentage^a^				
Spleen	BTBR	10.7±1.9	33.7±1.5^d^	0.52±0.28
	C57BL/6J	11.2±0.5	41.9±1.9	0.38±0.06
Lymph node	BTBR	20.4±2.0	27.0±2.1	0.93±0.43
	C57BL/6J	22.7±1.7	31.8±2.4	1.65±0.39
Peripheral blood	BTBR	13.4±1.0	48.0±2.8^c^	0.05±0.02^c^
	C57BL/6J	10.9±0.8	62.6±2.3	1.2±0.65
Number(×10^6^)^*b*^				
Spleen	BTBR	3.1±0.8	9.5±1.2	190.1±69.2
	C57BL/6J	2.7±0.5	8.8±1.6	80.2±7.3
Lymph node	BTBR	3.3±0.2^c^	4.8±0.9	109.9±40.8
	C57BL/6J	2.1±0.2	3.0±0.4	173.9±56.2
Peripheral blood	BTBR	1.55±0.48	5.47±1.00	0
	C57BL/6J	1.45±0.33	8.68±1.78	0

^*a*^Peripheral immune organs were obtained as described in FIGURE 5. The results are expressed as mean values ± SE of (*a*) percentages of gated CD45^+^ cells or (*b*) number of cells (blood ×10^3^/µl; organ ×10^6^ for CD8^+^ T cells and B cells and ×10^3^ for CD138^+^ plasma cells). Results are for whole spleen, lymph nodes - three mesenteric lymph nodes, and 1 ml of blood.

^*c*^Significant difference (*p*<0.001) between BTBR and C57BL/6J; normality passed.

^*d*^Significant difference (*p*<0.002) between BTBR and C57BL/6J by Mann-Whitney test.

**Table 4:** Proportions (%) of brain immune cell components.^a^

	Microglial cell	Dendritic cell	Mast cell
BTBR	94.4±0.9^b^	2.2±0.7	2.8±0.7
BCF1	94.8±1.7	2.4±0.7	2.3±1.2
CBF1	88.8±2.8	1.4±0.4	0.8±0.2
B6	86.7±3.0	0.9±0.2	1.0±0.2

^*a*^Brains were collected after perfusion from postnatal day 21 mice.

Flow cytometric analysis was applied to determine proportions of brain immune cell components among CD45^+^ cells. The results are expressed as mean values ± SEM.

^*b*^significantly different from that of B6 mice.

The types of immune cells (CD45^+^ hematopoietic cells) in the brain of BTBR, BCF, CBF, and B6 mice also were assayed (Table 4). As noted earlier, neuroinflammation could be a factor associated with the incidence of autism; thus, we assessed the percentage of brain immune cells known to be related to inflammation and pathogenesis, such as microglial cells, dendritic cells, and mast cells [41], [42], [45]. Two-way ANOVA (sex, strain) indicated a strain difference (F = 4.19, DF = 3, p<0.017) followed by one-way ANOVA (strain, p<0.014) and SNK *post-hoc* test, which indicated that the percentage of microglia in BTBR brains was different from that of CBF1 and B6 mice. Although the numbers of dendritic cells and FcεR1 expressing cells (presumably mast cells) in the brains of the BTBR mice were slightly greater than those of CBF1 and B6 mice, the differences were not significant by flow cytometric analysis, but the microglial cell percentage for BCF1 mice was significantly (*p*<0.05) higher than that for CBF1 or B6 males.

### Mast cells were more prominent in BTBR mice

**Figure 7:**
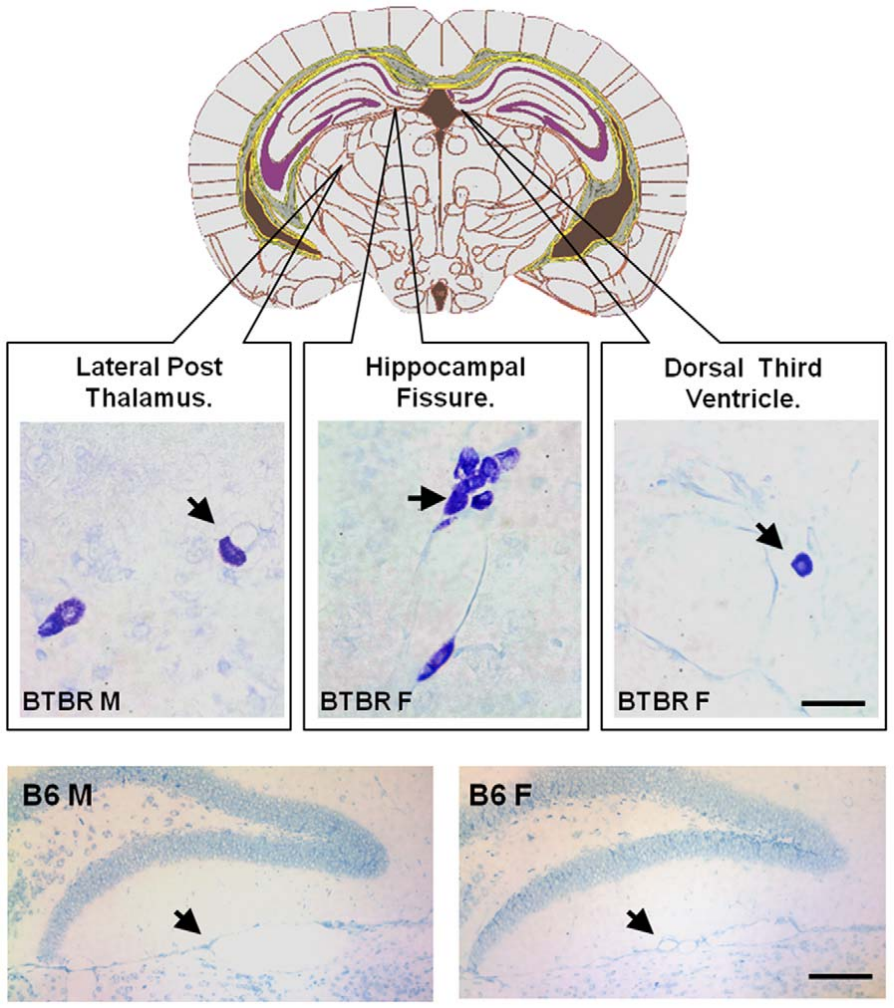
Mast cells in BTBR tissues. Diagram shows the level at which mast cells were apparent in the BTBR male and female brains (approximately Bregma −2.54 mm). Metachromatic staining of mast cells (purple) was clear on the orthochromatic (blue) background in tissue sections. Mast cells were visible at perivascular spaces in the lateral posterior thalamic nucleus, at the hippocampal fissure, and at the third ventricle adjacent to choroid plexus tissues, in BTBR male and female mice. Bar is 20 µm. Mast cells were not visible at the aforementioned regions in either male or female B6 mice, an example of hippocampal fissure tissues underneath dentate granule cells is shown from male and female cells. Only the orthochromatic (Blue) staining is visible. Bar is 60 µm.

Mast cells are rarely seen in healthy brains but have been reported to be sparsely present in the thalamus, hypothalamus, circumventricular organs, meninges and cerebral cortex of rodent brains [46]. We were unable to characterize a significant increase in the number of mast cells from cell suspensions of perfused BTBR brains by flow cytometry, we did observe a significant increase of mast cells. However, when forebrain, midbrain and cerebellar tissues encompassing Bregma +1.4 mm to Bregma −6.12 mm from male and female BTBR and B6 mice were stained with acidic toluidine blue, metachromatic mast cells were visible in the tissue from BTBR mice at the hippocampal fissure, lateral thalamic nuclei and third ventricle, but not at the caudate nucleus, cortex or cerebellum (Figure 7). The mast cells were perivascular and appeared to be entering brain parenchyma. There was a noticeable absence of mast cells in all of the aforementioned brain regions of the male and female B6 mice. Additionally, we noted increased numbers of mast cells in meningeal tissues from the BTBR mice.

### BTBR mice have increased numbers of IgG-secreting B cells but not increased numbers of total B cells

Although there were slightly lower numbers of B cells in BTBR mice, ELISPOT analysis indicated that there are more IgG-secreting B cells in BTBR spleens (239±47.9/10^5^ cells; N = 13) than in B6 spleens (60±54/10^5^ cells; N = 14).

### Expression of proinflammatory cytokines in brains of BTBR mice

**Figure 8:**
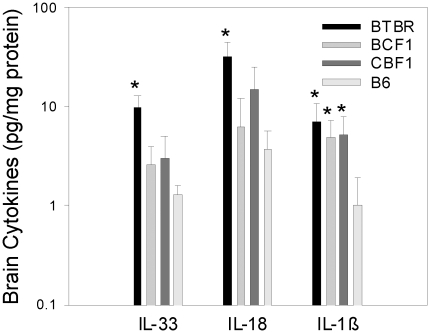
Brain expression of cytokines. Whole-brain homogenates were obtained from postnatal day 21 mice, as described. IL-18 and IL-33 were assayed by ELISA and all of the other cytokines were assayed by Luminex, including those not detected and therefore not shown. IL-33, IL-18, and IL-1β were significant based on one-way ANOVA on Ranks with p = 0.019, 0.007, and 0.013, respectively (*).

Despite a lack of consensus, a role of cytokine involvement, neuroinflammation, and/or skewed helper T cell reactivity has been suggested in the pathogenesis of autism [23], [41], [47], [48]. In addition, some cytokines have been reported to influence brain development, and abnormal expression of these cytokines has been considered to contribute to behavioral aberration in autistic subjects [23], [43], [49]. Therefore, we examined the expression of various cytokines in the whole brain (Fig. 8) and in individual brain regions (Table 5) of BTBR, B6, and F1 mice, focusing on proinflammatory cytokines such as IL-33, IL-18, IL-1β, IL-6, and TNFα. Since there were no gender differences for IgG levels, the results for males and females were pooled for the cytokines in the whole brain homogenates (Fig. 8). First, in terms of the level of cytokine expression in the whole brain, most of the statistically significant differences were found to exist between BTBR and B6 mice; no apparent difference was observed between BCF1 and CBF1 mice. Expression of three of the representative proinflammatory cytokines (IL-33, IL-18, and IL-1β) was higher in the BTBR mice than in B6 mice, and appeared intermediate in the BCF1 and CBF1 mice; the expression of IFNγ and of TNFα was low in BTBR brains (data not shown). The levels of IL-6 and IL-10 also were higher in the BTBR mice (0.46±0.07 for IL-6 and 0.77±0.40 for IL-10 pg/mg protein) than in the B6 mice (0.24±0.04, IL-6; 0.25±0.07, IL-10 pg/mg protein), but the differences were not significant. The expression levels of IL-12 or IL-2 differed little across the strains, except that the IL-2 level was lower BCF1 females (data not shown). Overall, IL-33, IL-18, and IL-1β were expressed to a greater extent in the whole brain of BTBR mice than in the whole brain of B6 mice.

**Table 5:** Levels of cytokines expressed in six brain regions.^a^

		Cytokine (pg/mg protein)								
Brain region	Strain	IL-33	IL-18	IL-1β	IL-6	IL-10	IFNγ	IL-12	IL-2	TNFα
**Cortex**	BTBR	16.6±5.2^b^	37.2±8.1	9.6±2.9	2.3±0.9	0.9±0.2	9.0±2.2	19.4±1.8	0.6±0.2	0.7±0.2
	BCF1	1.3±0.4	21.5±10.9	4.0±1.7	0.2±0.1	1.2±0.9	2.9±0.4	27.4±5.3	0.6±0.2	0.5±0.1
	CBF1	1.7±0.8	14.3±5.3	3.8±1.2	0.5±0.1	1.3±0.3	2.6±0.5	33.7±13.5	0.5±0.2	0.4±0.1
	B6	0.7±0.4^c^	11.3±6.7	1.9±0.9	0.4±0.1	0.6±0.2	10.2±2.1	19.8±4.0	0.9±0.1	1.2±0.2
**Striatum**	BTBR	11.4±2.4^b^	33.6±4.4^b^	9.4±3.4	2.4±0.7	1.1±0.3	10.6±3.8	37.4±8.8	1.4±0.4	1.3±0.3
	BCF1	2.3±1.1	13.3±8.2	6.9±3.3	0.6±0.2	1.5±0.5	3.5±0.7	49.4±13.3	1.3±0.4	1.1±0.2
	CBF1	2.7±1.2	18.8±7.6	7.9±1.8	0.6±0.2	1.9±0.6	3.9±0.8	58.9±23.4	1.0±0.4	0.7±0.2
	B6	1.8±0.4	11.7±5.5	2.3±1.3	0.3±0.1	0.7±0.2	5.3±1.2	35.1±8.1	1.0±0.2	1.5±0.3
**Hippocampus**	BTBR	10.8±2.5^b^	33.7±8.8^b^	8.0±2.7	3.4±1.2	1.8±0.7	13.0±5.2	27.0±6.3	0.7±0.2	1.1±0.3
	BCF1	2.4±0.8	9.0±3.8	3.9±1.5	0.2±0.1	0.8±0.3	2.8±0.3	22.7±6.8	0.4±0.2	0.9±0.3
	CBF1	3.0±1.3	14.4±6.0	3.6±1.5	0.5±0.2	1.0±0.4	4.0±0.7	31.0±11.0	0.8±0.3	0.6±0.2
	B6	1.0±0.3^c^	4.2±1.7^c^	1.1±0.5	0.4±0.1	0.5±0.2	2.9±1.2	18.5±4.5	0.5±0.2	1.1±0.3
**Hypothalamus**	BTBR	26.1±4.8^b^	32.3±5.4	16.2±3	4.0±1.4	2.6±0.7	13.3±4.4	83.2±23.2	2.4±0.7	2.7±0.7
	BCF1	6.6±2.7	13.9±2.1	11.4±7.3	1.1±0.2	3.5±1.2	6.7±1.2	98.5±23.0	2.4±0.8	2.3±0.6
	CBF1	9.4±4.0	19.6±6.3	15.1±5.1	1.8±0.3	4.0±1.8	7.3±1.0	96.3±36.3	2.6±0.8	1.8±0.4
	B6	3.8±1.0^c^	25.7±14.8	7.0±3.6	1.4±0.5	0.7±0.4	3.8±1.4	81.8±12.8	3.0±0.7	4.4±1.1
**Substantia nigra**	BTBR	38.8±6.9^b^	83.9±18.6	65.5±19.5^b^	11.5±3.4^b^	14.3±4.3^b^	44.3±11.9^b^	318±74^b^	11.8±4.0	9.8±2.3
	BCF1	17.4±12.2	15.2±5.5	30.6±13.7	4.2±1.0	6.7±1.4	7.9±2.3^d^	202±52	4.6±1.7	3.9±0.9
	CBF1	12.0±6.3	21.4±10.1	23.6±11.6	1.8±0.4^e^	7.1±3.0	10.8±2.8	84.2±35.3^e^	3.7±1.6	2.9±0.9
	B6	7.3±4.5	55.5±33.8	5.7±3.4^c^	1.4±0.6^c^	1.8±0.4^c^	11.8±3.1	83.8±28.3^c^	2.7±0.8	5.2±1.5
**Cerebellum**	BTBR	24.6±6.6^b^	33.2±6.9^b^	14.9±4.1^b^	2.4±0.7^b^	8.6±6.8	10.1±1.6^b^	24.8±5.6	1.0±0.2	1.1±0.3
	BCF1	2.1±0.9^d^	10.7±7.2	7.2±3.1	0.4±0.1^d^	3.6±1.1	2.8±0.4^d^	40.3±8.0	1.1±0.2	0.7±0.1
	CBF1	4.0±2.1	13.3±5.5	8.2±1.3	1.1±0.5	1.4±0.4	3.5±1.0	44.6±24.0	0.8±0.3	0.7±0.1
	B6	1.0±0.4^c^	6.4±3.3^c^	2.7±1.4	0.6±0.2	0.9±0.2	2.4±1.0^c^	51.0±17.9	1.1±0.3	1.6±0.4

^*a*^Each brain region was isolated from perfused postnatal day 21 mice as described in Table II.

The results are expressed as mean ± SEM.

^*b*^Cytokine levels in brain region of the mouse strains were significantly different by one-way ANOVA.

^*c*^ *post-hoc* test indicates that the regional cytokine level of B6 differs from that of BTBR.

^*d*^ *post-hoc* test indicates that the regional cytokine level of BCF1 differs from that of BTBR.

^*e*^ *post-hoc* test indicates that the regional cytokine level of CBF1 differs from that of BTBR.

The pattern of cytokine expression in each brain region (Table 5) was similar to the pattern seen for the whole brain (Fig. 8). In general, the expression levels of cytokines were highest in BTBR mice and lowest in B6 mice. Interestingly, there were significant strain differences for IL-33 in all brain regions; this was true only for IL-33. Surprisingly, the region with the most significant strain differences was the substantia nigra (SN); the SN is a very small brain region in which microglial cells and dopaminergic neurons are of higher proportion than most other brain regions [50]. In general, the SN and the cerebellum had the greatest differences between BTBR and B6 mice for the most cytokines. Notably, there were significantly lower levels of IFNγ and TNFα in whole brains of BTBR mice, in comparison with B6 whole brains, but this was not reproduced when the individual brain regions were investigated.

### Type-1/type-2 immune balance

**Figure 9:**
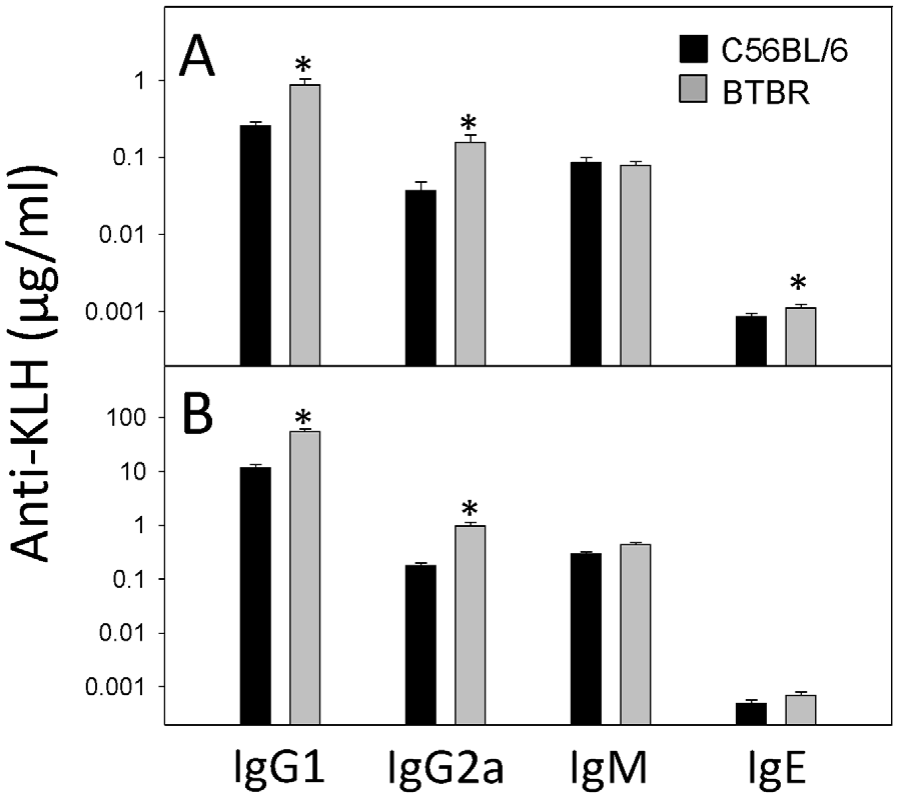
BTBR males have elevated anti-KLH serum levels. Primary (A) and secondary (B) serum Ab isotypes to KLH were measured by ELISA for the various strains. The primary and secondary responses were measured 7 days after immunization with 100 µg KLH without adjuvant; * indicates significant difference from that of the B6 level.

**Figure 10:**
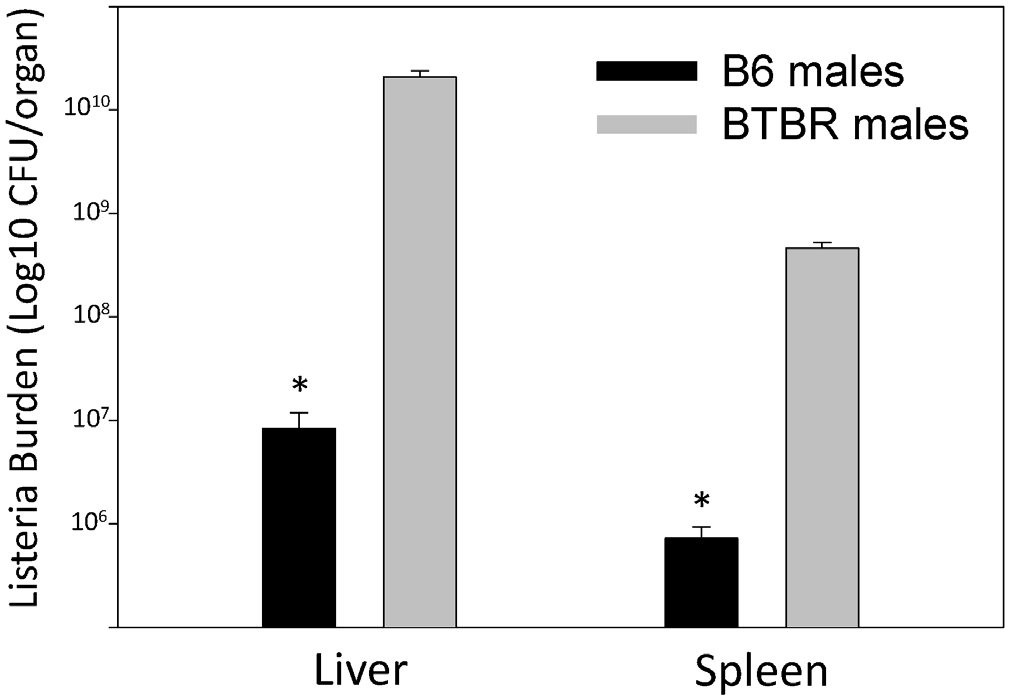
*Listeria monocytogenes* (LM) burden of infected mice. BTBR and B6 mice were assessed for LM (cfu/organ) in liver and spleen at threes days after infection; * indicates a significant difference between the two strains.

Since the BTBR mice expressed high levels of IgG and cytokines, we investigated humoral immunity to KLH and innate and cell-mediated immunity to LM of adult (pnd70) BTBR and B6 mice. To eliminate differences due to estrous cycle, only males were assessed. Primary and secondary anti-KLH levels were significantly higher in BTBR mice than the levels of B6 mice (Fig. 9). Immune defenses against LM were opposite of the BTBR and B6 responses observed for humoral immunity to KLH; BTBR mice had significantly less immunity and thus the cfu levels of LM were greater in livers and spleens. For BTBR mice, there were 2.1±0.3×10^10^ cfu LM in the liver and 4.6±0.5×10^8^ cfu LM in the spleen, whereas B6 mice had 8.4±3.5×10^6^ and 0.7±0.2×10^6^ cfu LM, respectively (Fig. 10). In previous studies with BALB/c mice, which are more susceptible than B6 mice to listeriosis, there were always lower cfu values than observed with BTBR mice. Since, innate and Th1 immune responses are responsible for defenses against LM, it seems that BTBR mice have poor innate and type-1 immunity. Interestingly, at 3 days after infection, the liver levels of IL-17, TGFβ, and IL-10 were significantly greater in BTBR mice than B6 mice (data not shown). The anti-KLH and LM immune responses suggest that BTBR mice have predominantly type-2 immunity. This suggestion is supported by the constitutively high levels of IgE in the sera of BTBR mice, which were significantly greater than those of B6 mice or the F1 offspring (15.4±1.3, BTBR; 1.6±0.2, B6; 3.4±0.7, CBF1; 2.8±0.3, BCF1; mean IgE µg/mL ± SEM). These results included pooled sera from males and females; however, most BTBR females had higher IgE levels than BTBR males (males, 11.9±1.5; females, 19.4±2.0). Since the serum IgE levels of the BTBR mice were elevated to an even greater extent than that of the IgG differences of BTBR and B6 mice, we assess, whether like IgG, IgE was elevated in the brains of the BTBR mice. Additionally, mast cells have been implicated in autism [51] and in the enhancement of immune cell entrance into the brain [52], and mast cells are triggered by IgE to release neurotransmitters and cytokines. Additionally, the BTBR mice do have more mast cells surrounding and in the brain. Thus, measurement of IgE presence in brain regions is of potential relevance to the heightened neuroinflammation of BTBR mice. Interestingly, the regional proportions of IgE (Table 6) were similar to those of IgG (Table 1) in the brains of the BTBR mice; however, the differences regarding the amounts of IgG and IgE in the brain regions of BTBR and B6 mice were not similar. All brain regions of BTBR mice had more IgG than those of B6 mice, but only the CTX, FCTX and HC of BTBR mice had more IgE. It is also interesting to note that the higher serum levels of IgE in BTBR females compared to BTBR males is observed as higher levels only in the STR, SN, HC, and CB.

**Table 6:** Levels of IgE in brain regions.^a^

			Level of IgE (ng/mg protein)^b^					
Strain	Sex	Cortex (CTX)^c^	Frontal Cortex (FCTX)^c^	Striatum (STR)^d^	Hippocampus (HC)^e^	Hypothalamus (HT)	Substantia nigra (SN)^d^	Cerebellum (CB)^d^
BTBR	Male	3.2±0.4	3.9±0.4	7.3±1.0	3.1±0.3	10.0±0.8	34.3±3.0	2.7±0.2
	Female	3.9±0.4	3.9±0.3	10.3±0.9	4.5±0.7	11.7±0.9	43.9±2.0	4.7±0.5
B6	Male	1.8±0.2	1.7±0.3	5.8±1.1	1.8±0.2	11.9±1.5	42.5±4.8	2.8±0.2
	Female	1.5±0.2	1.4±0.2	11.3±0.6	1.2±0.2	12.5±1.6	33.4±5.1	4.1±0.4

^*a*^Each brain region was isolated from perfused postnatal day 21 mice, and the homogenates were used for evaluation of IgE level present in each region. Number of mice was 4 for each sex and strain. The results are expressed as mean ± SEM.

^*b*^IgE levels in brain regions of the mouse strains were significantly different by three-way ANOVA with BTBR and B6 mice with SN and HT being the most significantly different regions.

^*c*^Two-way ANOVA indicates a strain difference; Bonferroni t-test indicates that BTBR differs from B6 (p<0.001).

^*d*^Two-way ANOVA indicates a sex difference; Bonferroni t-test indicates that males differ from females (p<0.001).

^*e*^Two-way ANOVA indicates strain and sex differences; Bonferroni t-test indicates that male and females differ within strain and strains differ (p<0.001).

## Discussion

**Figure 11:**
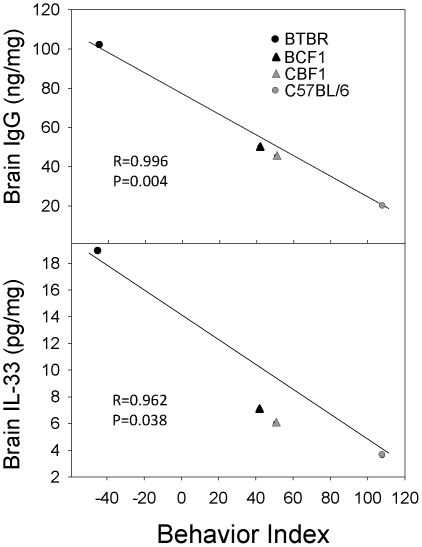
Correlation analysis of brain IgG and IL-33 of the strains with their behavior index.

In the present study, we have evaluated the basic immunological characteristics of BTBR mice. The BTBR strain has been reported to have abnormal behaviors that resemble autism [15]–[19], and our behavioral analysis supports the BTBR strain's previously described lack of sociability. The lack of sociability displayed by male and female BTBR mice was lost with the F1 offspring; however, the heterzygosity of the F1 strains results in an intermediate behavioral phenotype. The F1 offspring spent less time with the novel mouse and more time with the novel object than B6 mice. The intermediate behavioral phenotype is apparent with the behavior index (time with novel mouse minus time with novel object). With regard to the differential strain phenotypes, this time differential (behavior index) correlated with a number of immune parameters, especially IgG deposited in the brain and the amount of cytokines (e.g., IL-33) present in the brain (Fig. 11). Thus, the elevated IgG, which includes Abs deposited in the brain, and neuroinflammation inversely correlates with degree of sociablity. Functional alterations of the peripheral or central immune system have been studied in some individuals with ASD, and the implication of brain inflammation or generation of AutoAbs against brain antigens has been discussed, in the context of the background mechanisms of autism development [23], [29]. Beyond the documented autism-like behavior of BTBR mice [15]–[17], [26], [53], [54], our present investigation demonstrates that certain immunologic characteristics of BTBR mice compare well with the immune modulations reported from autistic subjects. Unlike B6 mice, an inbred strain with highly social behavior, 3 week old BTBR mice exhibited signs of neuroinflammation. BTBR mice expressed higher levels of brain-deposited IgG, and IgE, brain cytokines, and MHC class II expressing microglia than these levels in B6 mice; the peripheral level of CD4^+^ T cells also was higher in BTBR mice. The neuroinflammation could be due to the activated microglia or increased presence of mast cells, which were prominent within the meninges and brains of BTBR mice, especially at circumventricular organs, particularly the IIIrd ventricle, the hippocampal fissure, and perivascular spaces in the posterior lateral thalamus; these areas may play a role in the BTBR behavioral abnormalities.

BTBR mice also appear to have a predominant Th2 profile, in that they have high humoral immune responses to KLH, low immunity to LM, and significantly higher levels of serum IgE. If all of our data are taken together, BTBR inbred mice appear to be a strain highly susceptible to neuropathology, due to the elevated levels of cells, antibodies, and cytokines in their brains. Most interestingly, the two types of offspring from F1 crosses, BCF1 and CBF1 mice, have phenotypes intermediate between those of the parental BTBR and B6 mice, although the BCF1 mice seem to show slightly increased signs of neuroinflammation than do the CBF1 mice. These immunologic differences suggest that the BTBR strain could be a useful model to further study the developmental influences of immunity on aberrant behaviors.

There is overall consensus on male predominance for autism prevalence [55], [56]. Even though various genetic variations in X chromosome of autistic subjects have been reported, the relationship of such variance with pathogenesis of autism or ASD, especially with regard to a greater percentage of males having ASD (4∶1, male∶female, ratio), remains in doubt [57], [58]. Likewise, two quantitative trait loci found in X chromosome of BTBR strain were suggested to modulate the corpus callosum abnormalities associated with autism [59], but these chromosomal variations did not give rise to a gender difference in the anatomical brain defects [19]. Among autistic subjects, no striking difference between genders has been reported for immune abnormalities [23], [29]. Interestingly, for monozygotic male and female twins, concordance was reported to be 86 and 100%, respectively, for an ASD outcome, and for dizygotic male-male and female-male twins, the respective concordance values were 40% and 20% [60]. Thus, yet undefined gender influences appear to exist. Gender influences were not evident in our analysis of the central and peripheral levels of IgG in BTBR mice; there also were no gender differences with regard to expression of proinflammatory cytokines and immune cell composition. Most published studies of the autism-like behaviors of BTBR mice have employed male mice only; no published data have indicated a significant behavioral difference between male and female BTBR mice. Therefore, the current assumption seems to be that sex-linked genetic differences do not influence the development of proinflammatory and autoimmune-prone immunologic characteristics of BTBR mice. Nevertheless, some genetic contribution, especially maternal nuclear or mitochondrial genes likely contribute to the immune modulations, given that BCF1 mice born of BTBR dams showed higher autoreactive IgG level, microglial cell percentage, and expression of proinflammatory cytokines than did B6 mice. Genetically, the two F1 strains differ in terms of mitochondrial inheritance as well as X- and Y-linked genes and potentially early maternal epigenetic influences.

With respect to maternal influence on the autistic phenotype of offspring, two published experimental approaches provide a hint as to the role of maternal IgG against fetal brain antigens, in the induction of behavioral alterations after birth [27], [28]. Both of the studies used serum IgG purified from the blood of mothers of autistic children, which was injected intravenously into pregnant Rhesus monkeys or was injected intraperitoneally into pregnant B6 mice. Reactivity of serum IgG against fetal brain proteins was confirmed through Western blot analysis or immunohistochemical staining. Rhesus monkey infant offspring and adolescent/adult B6 offspring that had been prenatally exposed to IgG from mothers of autistic children displayed higher levels of stereotypic and hyperactive motor activity than did controls [27]; the prenatally exposed mice, as adults, displayed less social interactions with strangers than did controls [28]. The two studies provided clear evidence for involvement of antibody in the development of autistic behaviors in offspring. Our present findings indicate that the level of anti-brain IgG is higher in BTBR or BCF1 mice born of BTBR dams than in B6 or CBF1 mice born of B6 dams. Analyses in the literature of serum from autistic children or mothers of autistic children have detected Abs against various CNS Ags, including myelin basic protein, serotonin receptor, glial fibrillary acidic protein, brain-derived neurotrophic factor, and heat shock proteins [24]–[26], [33], [34]. A role of AutoAbs in the pathogenesis of autism is not proven, nor is the involvement of autoimmune mechanisms in ASD. However, clearly, identifying the specificities and etiologic roles of AutoAbs should be further investigated.

Cytokines affect the development of neuronal or glial cells, as well as behavioral phenotypes [43], [61]. In autistic subjects, immune alterations, particularly cytokine modulations, that affect the pathogenesis of autism development, are not agreed upon [23], [62]. Vargas et al. [42] provided evidence for an ongoing process of neuroinflammation in the CNS of their autistic subjects. Macrophage chemoattractant protein-1, a proinflammatory cytokine, was the typical cytokine prominently expressed in both brain and cerebrospinal fluid of autistic patients. Marked activation of microglial cells and astrocytes was also evident across the brain regions, especially in the cerebellum. An anti-inflammatory cytokine, tumor growth factor-β1, was simultaneously elevated in brain regions indicative of chronic neuroinflammation. In addition to the usual analysis of IL-1β and TNFα, IL-33 and IL-18 were assayed in our study. Overall, levels of several cytokines, including IL-33, IL-18, IL-1β, and IL-6, were elevated, whereas IFNγ and TNFα were decreased in the brains of PND21 BTBR mice, compared to brains of B6 or CBF1 pups. IL-33, IL-18, and IL-1β share many traits: synthesis as pro-forms lacking activity, production of mature/active forms through caspase-1 cleavage, secretion via endoplasmic reticulum/Golgi-independent pathway, and biological activity for inflammation [63]–[66]. Glial cells are a major source of IL-33, IL-18, and IL-1β production in the brain. The percentage of microglial cells was higher in the BTBR mice than in B6 mice, perhaps related to the higher levels of these cytokines in the brains of the former strain. Furthermore, when we compared the mean fluorescence intensity of MHC-II expression on brain microglial cells, the expression intensity was higher (although the difference was not significant) in the BTBR (670±204) than in the B6 mice (498±314). The greater MHC-II expression, a phenomenon also observed in Parkinson's disease [67], suggests an elevated state of activation of microglia in the BTBR mice. Among the above three proinflammatory cytokines, IL-1β is the only one to have been reported in previous investigations of immune alterations in autistic subjects [23], [62]; the level of IL-1β from stimulated or unstimulated peripheral mononuclear cells *in vitro* was higher in autistic children than in controls. Elevation of IL-18 has been reported for rodents and humans with psychiatric disorders [65]. Among the brain regions that we examined, the cerebellum was one that demonstrated significantly higher expression of both IL-33 and IL-18 in BTBR mice than in B6 mice; the cerebellum could thus be a “hot” region for neuroinflammation in the BTBR mice and for ASD in humans. Loss of Purkinjie cells has been frequently observed in the cerebellum, and neuronal degeneration and glial cell activation have predominantly been found in the cerebellum of autistic patients [39], [68].

The Th1-Th2 paradigm, that is immune skewing toward type-1 cell-mediated immunity (interferon-gamma promoted immunity) *vs.* type-2 humoral immunity (IL-4, IL-5 and IL-13 promoted immunity), may not be properly applicable to immune alterations in autism. Conflicting results have been derived from human studies on a Th1 or Th2 shift: several investigations reported Th1 skewedness, but other studies suggested a Th2 shift [23], [62]. Here, we found a substantial downregulation of the Th1 response in BTBR mice, as compared to B6 mice; IL-12 and IFNγ expression was similar or lower, whereas IL-33 and IL-10, cytokines promoting Th2-mediated response [22], [64], were elevated. The greater IL-6 expression in the brain of BTBR mice may be worth noting since IL-6 is involved in sickness behavior and also in modulation of brain development (probably mediated by neuronal inflammation) [69], [70]. In addition, IL-6 has been reported to mediate the behavioral abnormalities of adult offspring from dams that experienced maternal immune activation during pregnancy [49]. Since type-2 immunity appears to predominate in BTBR mice and IL-33, a type-2 immune promoter, is the only cytokine significantly elevated in all brain regions, IL-33 may be especially important to consider for its affects on behavior, in that it has been suggested that IL-33-producing glia are critical regulators of innate immune responses in the CNS [71].

The existence of an animal model will be very valuable in the investigation of mechanistic pathways and therapeutic interventions of psychiatric diseases, especially for neurodegenerative or neurodevelopmental disorders. The present study is the first report of the involvement of neuroinflammation, accompanied by elevation of IgG and IgE and IgG-secreting B cells, in BTBR mice, a strain with abnormal behaviors. Considering the elevated amount of IgG accumulated in the brain and serum anti-brain antibodies of BTBR mice, specific brain Ag(s) need to be identified, and the involvement of humoral immunity in the aberrant behaviors of the BTBR mice needs to be explored. The genetic basis for the constitutively high levels of IgG and IgE also need to be investigated. Although higher levels of several proinflammatory cytokines, including IL-33, IL-18, and IL-1β, were shown in BTBR in the present study, brain cellular sources of these cytokines need to be delineated. It also is necessary to evaluate the individual contribution of each cytokine to the development of the aberrant BTBR behaviors, as well as the cross-talk between proinflammatory cytokines and certain anti-inflammatory cytokines, such as TGFβ1. Since BCF1 mice (born of BTBR dams, sired by B6), especially the males, demonstrated immunologic characteristics closer to BTBR mice than did the CBF1 mice, these combinations may be useful for dissection of the genetic and environmental influences on development of the aberrant behaviors.

## Materials and Methods

### Mice

BTBR breeder mice were kindly provided by Dr. Valerie J. Bolivar (Wadsworth Center, New York State Department of Health). BTBR and B6 mice also were purchased from Jackson Laboratories (Bar Harbor, ME), and BALB/cByJ and BALB/c- *scid* mice (mice lack B cells and T cells and thus have no IgG) were obtained from the Wadsworth Center's Animal Production Unit. BTBR and B6 mice are both H-2^b^. Mice were housed in sterile laminar flow cages in a specified pathogen free facility of the Wadsworth Center and were maintained on autoclaved food and water. All of our animal maintenance, breeding, and experimental procedures were approved by the Wadsworth Center's IACUC, Protocol # 09-278. F_1_ mice were derived from BTBR females×B6 males (BCF1) or B6 females×BTBR males (CBF1). All of the mice used were sacrificed at pnd21 or after behavioral analysis at pnd70. All of the experimental data were obtained from at least three separate litters for each strain and both genders.

### Preparation of brain tissue homogenates for IgG or cytokine measurement

Whole brain or various brain regions (cortex, striatum, hippocampus, hypothalamus, substantia nigra, and cerebellum) were collected from pnd21 mice following intracardial perfusion with warm phosphate-buffered saline (PBS; 50 ml/mouse). Each brain was dissected with microdissection forceps and scissors. The brain tissues were homogenized in the extraction buffer containing 20 mM Tris, 100 mM NaCl, 2 mM Na_2_EDTA, 1% NP-40, and protease inhibitor cocktail (Sigma, St. Louis, MO), followed by sonication for 10 min. The supernatants were collected following 30 min centrifugation (16,000 *g*) at 4°C, and kept frozen before use. The homogenate protein concentration was determined with BCA protein assay kit (Pierce, Rockford, IL).

### IgG ELISA

The level of total IgG in serum or brain homogenates was determined by a sandwich ELISA using goat anti-mouse IgG Fc (Pierce, Rockford, IL) as a capture Ab and HRP-goat anti-mouse IgG as a detection Ab (Sigma). Brain homogenates and sera were diluted 1/100 and 1/100,000, respectively, with PBS.

Presence of brain-reactive IgG in mice sera was determined as follows [72]. SCID mouse whole brain or brain regional proteins were coated (10 µg/well) in the 96-well plate overnight at 4°C, and after 3× washing, the plate was blocked with 5% BSA-PBS for 2 h. SCID brains were used so as to ensure the absence of IgG in the brain homogenates. Sera from pnd21 mice were added into the wells, and incubated overnight at 4°C. After 6× washing, HRP-goat anti-mouse IgG detection Ab was added and incubated for 2 h at room temperature. The plates were washed 6×, and the BD Pharmingen™ TMB substrate solution (San Diego, CA) was added for color development. Absorbance was measured at 450 nm, with 570 nm as the reference wavelength. SCID serum was used as a negative control.

### ELISPOT analysis of immunoglobulin-secreting cells

MultiScreen Filtration plate (Millipore, Billerica, MA) was pre-wetted with 50 µL of 70% ethanol in sterile PBS per well. After 2× wash with sterile PBS, the plate was coated with goat anti-mouse IgG γ chain (Mabtech, Cincinnati, OH) as capture Ab (2 µg/100 µL/ well) in sterile PBS at 4°C overnight. The plate was washed 5× with PBS, and then RPMI 1640 medium containing 5% BSA was used to block the plate at 37°C for 2 hr. The blocking medium was discarded, and 10^5^ splenic cells in 100 µL of complete RPMI 1640 medium were added to each well and incubated at 37°C with 7% CO_2_, 5% O_2_, 88% N_2_, and high humidity for 24 hr. The cells were then discarded, and the plate was washed 6× followed by addition of biotinylated goat anti-mouse IgG (0.2 µg/100 µL/well in PBS containing 0.05% tween 20 and 1% BSA; Mabtech) and streptavidin-ALP ( 100 µL/ well; 1∶1000 diluted with PBS; Mabtech). After additional washings, the plate was incubated with the substrate BCIP/NBT for 15 min at room temperature. Then the plate was washed extensively with water and dried in the dark. The next day the plate was read with an ImmuoSpot analyzer (Cellular Technology, Shaker Heights, OH).

### Cytokine measurement

Nine cytokines (IL-33, IL-18, IL-1β, IL-6, IL-10, IFNγ, IL-12, IL-2, TNFα) were assayed for expression in brain homogenates from pnd21 pups. IL-33 was quantified with the DuoSet ELISA set (R&D, Minneapolis, MN). IL-18 was assayed using rat anti-mouse IL-18 Ab and biotinylated rat anti-mouse IL-18 Ab, as a paired capture and detection Ab (R&D). The levels of the other cytokines were evaluated with a Fluorokine MAP mouse multianalyte profiling kit (R&D) and analysis with a Luminex 100 (Austin, TX). The respective lower limits of detection were 1.21, 0.2, 0.078, 4.0, 1.81, 0.65, and 0.01 pg/ml, for IL-1β, IL-6, IL-10, IFNγ, IL-12, IL-2, and TNFα, respectively.

### Flow cytometric analysis

Single cell suspensions were prepared from spleens or mesenteric lymph nodes as described elsewhere [73], [74]. Three mesenteric lymph nodes were collected from each mouse. Mononuclear cell populations in the spleens or lymph nodes were determined by four color fluorescence-activated cell sorting (FACS) with a FACSCalibur flow cytometer (BD Biosciences). Anti-CD45-PerCP, anti-CD3-APC, and anti-CD4-FITC Abs were used for helper T cell phenotyping, and instead of anti-CD4-FITC, anti-CD8-FITC or anti-CD19-FITC, anti-CD5-APC Ab was used for cytotoxic T cell or B cell phenotyping, respectively. .Anti-CD40-PE and anti-I-A-PE Abs also were used for B cells. In addition to anti-CD45-PerCP and anti-CD3-APC Abs, anti-CD19-FITC and anti-CD138-PE Abs were added for enumeration of a plasma cell population. Absolute numbers and percentages of these cell populations in whole blood were determined with use of BD TruCOUNT™ tubes (50 µl blood/tube).

Preparations of brain single-cell populations containing microglial cells, dendritic cells, and mast cells were performed as described [75], [76], with a modification. In brief, after perfusion with PBS, brains were removed, incubated with liberase (0.2 mg/brain, Roche, Indianapolis, IN) for digestion, and minced; use of liberase for obtaining single cell suspensions of brain cells was previously described [77]. The homogenized brain samples were passed through 55 µm diameter nylon mesh. The resulting suspensions were centrifuged at 250 *g* for 8 min, and the pellet was resuspended in 40% Percoll (GE Healthcare, Piscataway, NJ) solution, followed by centrifugation at 800 g for 30 min. The resulting pellet was washed with 40 ml of 3% FBS-PBS through centrifugation, and then resuspended in PBS with 0.1% sodium azide. Four-color FACS was applied to determine the proportions of the various brain cell subpopulations by a FACSCanto (BD Biosciences) flow cytometer. Anti-CD45-APC-Cy7, anti-CD11b-PE-Cy7 (BD Biosciences), and anti-F4/80-Alexa Fluor 488 (Serotec, Oxford, UK) Abs were used for identification of microglial cells. Instead of anti-F4/80-Alexa Fluor 488 Ab, anti-CD11c-FITC or anti-FcεR1-FITC (eBioscience, San Diego, CA) Ab was used for enumeration of brain dendritic cells or mast cells, respectively. TO-PRO-3 (Invitrogen, Carlsbad, CA) was used to define viable cells.

### Immunohistochemistry

For the evaluation of IgG deposition in the brain, we utilized perfused brains; 200 µm sections were washed in wells of a 24-well culture plate with 6 washes with PBS to remove IgG not specific to a brain antigen. The sections were then fixed 4% paraformaldehyde in PBS for 1 hr at room temperature (RT) followed by 3 washes with PBS. The sections were then blocked with 3% goat serum in PBS for 30 min at RT and stained with 488-conjugated chicken anti-mouse IgG (1∶300 diluted with 3% goat serum in PBS) overnight at 4°C. The sections were washed 6 times, placed on charged slides and assayed by fluorescence microscope.

### Toluidine Blue staining and Digital Image Acquisition

Mice were perfused with PBS followed by 4% PFA, then sacrificed by CO_2_ asphyxiation. Brains were immediately removed, rinsed in saline and then immersion-fixed in 4% paraformaldehyde for 48 hr at 4°C. Fixed brain tissues were paraffinized using a Tissue Tek processor, and sectioned at 6 µm using a rotary microtome. In this study, tissue sections at the level of the forebrain caudate nucleus (Bregma +1.4 mm), rostral hippocampus (Bregma −1.3 mm), medial hippocampus (Bregma −2.5 mm) and cerebellum (Bregma −6.12 mm) were stained with toluidine blue to ascertain whether there were region and strain differences in mast cell density and morphology between BTBR and B6 mice. Briefly, for mast cell identification, tissue sections were cleared in xylenes, prior to rehydration through graded alcohols to distilled water. Toluidine blue staining solution was freshly prepared, and comprised 5 ml of toluidine stock solution (1 g toludine blue – Sigma in 100 ml of 70% alcohol) with 45 ml of 1% Sodium Chloride acidified solution at pH 2.2. Sections were immersed in toluidine blue for 10 sec then rinsed 3 times in distilled water. Sections were then dehydrated through graded alcohols, cleared in xylenes prior to mounting with DPX (sigma) and coverslipping. For digital image acquisition, regions of interest were identified through comparison with a sterotaxic mouse brain atlas of Paxinos and Franklin [78]. Digital photo-micrographs were generated using a Nikon (50i) microscope using 10× and 60× lens coupled to a CCD camera with image grabber software. Mast cells were clearly visible as metachromatic purple color on an orthochromatic blue background.

### Blood Brain Barrier (BBB) permeability test

The BBB test for pnd21 mice was performed through examination of vascular leakage of Evans blue (EB) dye [79]. EB dye solution (50 µg/g body weight) was intraperitoneally injected into mice, and after 5 h, the mice were anesthetized with CO_2_ gas, and cardiac blood was collected for plasma separation. Brains were removed after PBS perfusion and weighed. Formamide (0.5 ml/brain, Sigma-Aldrich) was added to the tube containing brain, for extraction of EB dye from brain. The tubes were centrifuged at 16,000 *g* for 15 min for collection of supernatants after 72 h incubation with formamide. Optical density (OD) of the supernatants or plasma was measured at 620 nm by an absorbance microplate reader (Bio-Tek, Winooski, VT). The OD of pure formamide solution was subtracted as a background absorbance from each sample's OD (sample's adjusted OD). The adjusted ODs of brain samples were again adjusted to reflect weight differences among brain samples (adjusted OD). Finally, the BBB permeability index was calculated via division of the adjusted OD of each brain sample by the adjusted OD of the matching plasma sample followed by multiplication by 10.

### Social interaction

Critical to the validity of any biochemical, immunological, or molecular analyses for the autism-like assessments is the behavioral test [15], [80]. The sociability task measures the tendency of the subject mouse to approach the stimulus (novel) mouse, as well as the time spent with the mouse; these sessions include specific behaviors such as sniffing and licking. The novel mouse (BALB/cByJ) used is genetically different from the test mice, but the same gender, and is used only for one test per day. Behaviors of test mice were assayed at 8–10 wk of age. The stimulus mouse is enclosed inside a small circular wire cage. Thus, the stimulus mouse can provide visual, olfactory, auditory and some physical contact cues. An identical empty wire cage is placed in the other adjacent chamber. At the end of the habituation period, the dividers are removed so that the subject mouse can explore either of the adjacent chambers. The testing session is 10 min long. If the subject mouse is “sociable” it will spend more time in the chamber with the stimulus mouse. If not, it will either spend more time in the other adjacent chamber or remain in its central chamber. The calculated “behavior index” is the time spent with the mouse minus the time spent with the object.

### Anti-KLH response

Mice were immunized with KLH (100 µg in 200 µl saline). One week later, blood was collected by retro-orbital phlebotomy for sera and the mice were boosted with 100 µg KLH in 200 µl saline. Blood was again collected after 1 week. The sera were assayed for IgM anti-KLH, IgG1 anti-KLH, IgG2a anti-KLH, IgE anti-KLH, and total IgE by a standard ELISA assay, as described previously [81]. Briefly, KLH (10 µg/ml) in 0.1 M carbonate pH 9.5 (8.4 g NaHCO_3_, 3.56 g Na_2_CO_3_, in 1 L) was coated onto ELISA plates overnight at 4°C. After blocking with PBS plus 1% BSA, a serial dilution of the internal control (mouse IgG2a anti-KLH; Sigma) or serum was added. Biotinylated detection mAbs (biotin anti-mouse IgG1, IgG2a, or IgM from BD Science) were applied followed by Avidin-peroxidase (PharMingen) and substrate. The plates were read using an ELISA reader (EL310, Bio-Tek, Burlington, VT) at 450 nm. For measurement of KLH-specific IgE, 2 µg/ml purified anti-mouse IgE (PharMingen) was coated overnight at 4″C. After blocking with PBS plus 1% BSA, a serial dilution of the internal control (mouse IgE, PharMingen) and serum was added. Then, KLH (10 µg/ml) was added into sample wells only. After 2-hr incubation at room temperature, mouse IgG2a anti-KLH, followed by Biotin anti-mouse IgG2a, avidin-peroxidase was applied. For the internal control development, 2 µg/ml biotin anti-mouse IgE (PharMingen) and avidin-peroxidase were used. The same method was utilized for color development and for reading of the plates. For total IgE, the OptEIA Mouse IgE Set (PharMingen) was employed. The protocol provided by the manufacturer was used.

### Host resistance against *Listeria monocytogenes*

Host resistance against *Listeria monocytogenes* (LM) was measured as previously described [82]. Briefly, adult mice were injected intravenously with 10^5^ viable LM. Mice were weighed daily for assessment of sickness behavior, measured as lack of eating and drinking leading to weight loss. At day three after infection (the peak response), livers and spleens were aseptically harvested for assessment of bacterial burden by enumeration of LM colony forming units (CFU); serial dilutions of organ homogenates are plated in quadruplicate on blood-agar plates. Bacterial burdens are expressed as CFU per organ.

### Statistical analysis

The data were expressed as mean values ± SEM, and initially evaluated by three-way, two-way, or one-way ANOVA (gender, strain); however, since there were no significant gender differences, some of the data were further evaluated for normal distributions by one-way ANOVA and *post-hoc* tests. Differences with *p*<0.05 were considered significant. Analyses utilized SigmaPlot 11.

## References

[1] Goines P, Van de Water J (2010). The immune system's role in the biology of autism.. Curr Opin Neurol.

[2] Cohly HH, Panja A (2005). Immunological findings in autism.. Int Rev Neurobiol.

[3] Pardo CA, Vargas DL, Zimmerman AW (2005). Immunity, neuroglia and neuroinflammation in autism.. Int Rev Psychiatry.

[4] Vojdani A, Mumper E, Granpeesheh D, Mielke L, Traver D (2008). Low natural killer cell cytotoxic activity in autism: the role of glutathione, IL-2 and IL-15.. J Neuroimmunol.

[5] Castellani ML, Conti CM, Kempuraj DJ, Salini V, Vecchiet J (2009). Autism and immunity: revisited study.. Int J Immunopathol Pharmacol.

[6] Heuer L, Ashwood P, Schauer J, Goines P, Krakowiak P (2008). Reduced levels of immunoglobulin in children with autism correlates with behavioral symptoms.. Autism Res.

[7] Magalhães ES, Pinto-Mariz F, Bastos-Pinto S, Pontes AT, Prado EA (2009). Immune allergic response in Asperger syndrome.. J Neuroimmunol.

[8] Stern L, Francoeur MJ, Primeau MN, Sommerville W, Fombonne E (2005). Immune function in autistic children.. Ann Allergy Asthma Immunol.

[9] Theoharides TC (2009). Autism spectrum disorders and mastocytosis.. Int J Immunopathol Pharmacol.

[10] Singh VK (1996). Plasma increase of interleukin-12 and interferon-gamma. Pathological significance in autism.. J Neuroimmunol.

[11] Croonenberghs J, Bosmans E, Deboutte D, Kenis G, Maes M (2002). Activation of the inflammatory response system in autism.. Neuropsychobiology.

[12] Vargus DL, Nascimbene C, Krishnan C, Zimmerman AW, Pardo CA (2005). Neuroglial activation and neuroinflammation in the brain of patients with autism.. Ann Neurol.

[13] Molloy CA, Morrow AL, Meinzen-Derr J, Schleifer K, Dienger K (2006). Elevated cytokine levels in children with autism spectrum disorder.. J Neuroimmunol.

[14] Li X, Chauhan A, Sheikh AM, Patil S, Chauhan V (2009). Elevated immune response in the brain of autistic patients.. J Neuroimmunol.

[15] Moy SS, Nadler JJ, Young NB, Perez A, Holloway LP (2007). Mouse behavioral tasks relevant to autism: phenotypes of 10 inbred strains.. Behav Brain Res.

[16] Bolivar VJ, Walters SR, Phoenix JL (2007). Assessing autism-like behavior in mice: variations in social interactions among inbred strains.. Behav Brain Res.

[17] McFarlane HG, Kusek GK, Yang M, Phoenix JL, Bolivar VJ (2008). Autism-like behavioral phenotypes in BTBR T+tf/J mice.. Genes Brain Behav.

[18] Wohr M, Roullet FI, Crawley JN (2010). Reduced scent marking and ultrasonic vocalizations in the BTBR T+tf/J mouse model of autism.. Genes Brain Behav.

[19] Wahlsten D, Metten P, Crabbe JC (2003). Survey of 21 inbred mouse strains in two laboratories reveals that BTBR T+tf/tf has severely reduced hippocampal commisure and absent corpus callosum.. Brain Res.

[20] Barnea-Goraly N, Kwon H, Menon V, Eliez S, Lotspeich L (2004). White matter structure in autism: preliminary evidence from diffusion tensor imaging.. Biol Psychiatry.

[21] Alexander AL, Lee JE, Lazar M, Boudos R, DuBray MB (2007). Diffusion tensor imaging of the corpus callosum in autism.. Neuroimage.

[22] Steinman L (2004). Elaborate interactions between the immune and nervous system.. Nat Immunol.

[23] Ashwood P, Wills S, Van de Water J (2006). The immune response in autism: a new frontier for autism research.. J Leukoc Biol.

[24] Braunschweig D, Ashwood P, Krakowiak P, Hertz-Picciotto I, Hansen R (2008). Autism: maternally derived antibodies specific for fetal brain proteins.. Neuro Toxicology.

[25] Singer HS, Morris CM, Gause CD, Gillin PK, Crawford S (2008). Antibodies against fetal brain in sera of mothers with autistic children.. J Neuroimmunol.

[26] Croen LA, Braunschweig D, Haapanen L, Yoshida CK, Fireman B (2008). Maternal mid-pregnancy autoantibodies to fetal brain protein: the early markers for autism study.. Biol Psychiatry.

[27] Martin LA, Ashwood P, Braunschweig D, Cabanlit M, Van de Water J (2008). Sterotypies and hyperactivity in rhesus monkeys exposed to IgG from mothers of children with autism.. Brain Behav Immun.

[28] Singer HS, Morris C, Gause C, Pollard M, Zimmerman AW (2009). Prenatal exposure to antibodies from mothers of children with autism produces neurobehavioral alterations: a pregnant dam mouse model.. J Neuroimmunol.

[29] Moscavitch S-D, Szyper-Kravitz M, Shoenfeld Y (2009). Autoimmune pathology accounts for common manifestations in a wide range of neuro-psychiatric disorders: the olfactory and immune system interrelationship.. Clin Immunol.

[30] Wills S, Cabanlit M, Bennet J, Ashwood P, Van de Water J (2007). Autoantibodies in autism spectrum disorders (ASD).. Ann N Y Acad Sci.

[31] Croen LA, Goines P, Braunschweig D, Yolken R, Yoshida CK (2008). Brain-derived neurotrophic factor and autism: maternal and infant peripheral blood levels in the early markers for autism (EMA) study.. Autism Res.

[32] Cabanlit M, Wills S, Goines P, Ashwood P, Van de Water J (2007). Brain-specific autoantibodies in the plasma of subjects with autistic spectrum disorder.. Ann N Y Sci.

[33] Kirkman NJ, Libbey JE, Sweeten TL, Coon HH, Miller JN (2008). How relevant are GFAP autoantibodies in autism and Tourette syndrome?. J Autism Dev Disord.

[34] Libbey JE, Coon HH, Kirkman NJ, Sweeten TL, Miller JN (2008). Are there enhanced MBP autoantibodies in autism?. J Autism Dev Disord.

[35] Hoekstra PJ, Horst G, Limburg PC, Troost PW, van Lang N (2003). Increased seroactivity in tic disorder patients to a 60 KDa protein band from a neuronal cell line.. J Neuroimmunol.

[36] Yang M, Clarke AM, Crawley JN (2009). Postnatal lesion evidence against a primary role for the corpus callosum in mouse sociability.. Eur J Neurosci.

[37] Petkova SB, Yuan R, Tsaih SW, Schott W, Roopenian DC (2008). Genetic influence on immune phenotype revealed strain-specific variations in peripheral blood lineages.. Physiol Genomics.

[38] Kustova Y, Grinberg A, Basile AS (1999). Increased blood-brain barrier permeability in LP-BM5 infected mice is mediated by neuroexcitatory mechanisms.. Brain Res.

[39] Morrey JD, Olsen AL, Siddharthan V, Motter NE, Wang H (2008). Increased blood-brain barrier permeability is not a primary determinant for lethality of West Nile virus infection in rodents.. J Gen Virol.

[40] Beauchesne É, Desjardins P, Hazell AS, Butterworth RF (2009). Altered expression of tight junction proteins and matrix metalloproteinases in thiamine-deficient mouse strain.. Neurochem Intl.

[41] Blaylock RL, Strunecka A (2009). Immune-glutamatergic dysfunction as a central mechanism of the autism spectrum disorders.. Curr Med Chem.

[42] Vargas DL, Nascimbene C, Krishnan C, Zimmerman AW, Pardo CA (2004). Neuroglial activation and neuroinflammation in the brain of patients with autism.. Ann Neurol.

[43] Bilbo SD, Schwarz JM (2009). Early-life programming of later-life brain and behavior: a critical role for the immune system.. Front Behav Neurosci.

[44] Torrente F, Ashwood P, Day R, Machado N, Furlano RI (2002). Small intestinal enteropathy with epithelial IgG and complement deposition in children with regressive autism.. Mol Psychiatry.

[45] Theoharides TC, Kempuraj D, Tagen M, Conti P, Kalogeromitros D (2007). Differential release of mast cell mediators and pathogenesis of inflammation.. Immunol Rev.

[46] Michaloudi HC, Papadopoulos GC (1999). Mast cells in the sheep, hedgehog and rat forebrain.. J Anat.

[47] Hendren RL, Bertoglio K, Ashwood P, Sharp F (2009). Mechanistic biomarkers for autism treatment. Med.. Hypotheses.

[48] Ashwood P, Enstrom A, Krakowiak P, Hertz-Picciotto I, Hansen RL (2008). Decreased transforming growth factor beta 1 in autism: a potential link between immune dysregulation and impairement in clinical behavioral outcomes.. J Neuroimmunol.

[49] Smith SEP, Li J, Garbett K, Mirnics K, Patterson PH (2007). Maternal immune activation alters fetal brain development through interleukin-6.. J Neurosci.

[50] Beach TG, Sue LI, Walker DG, Lue LF, Connor DJ (2007). Marked microglial reaction in normal aging human substantia nigra: correlation with extraneural neuromelanin pigment deposits.. Acta Neuropathol.

[51] Theoharides TC, Angelidou A, Alysandratos KD, Zhang B, Asadi S (2010). Mast cell activation and autism.. Biochim Biophys Acta.

[52] Sayed BA, Christy AL, Walker ME, Brown MA (2010). Meningeal mast cells affect early T cell central nervous system infiltration and blood-brain barrier integrity through TNF: a role for neutrophil recruitment?. J Immunol.

[53] Benno R, Smirnova Y, Vera S, Liggett A, Schanz N (2009). Exaggerated responses to stress in the BTBR T+tf/J mouse: an usual behavioral phenotype.. Behav Brain Res.

[54] Scattoni ML, Gandhy SU, Ricceri L, Crawley JN (2008). Unusual repertoire of vocalizations in the BTBR T+tf/J mouse model of autism.. PLoS ONE.

[55] Bertrand J, Mars A, Boyle C, Bove F, Yeargin-Allsopp M (2001). Prevalence of autism in a United States population: The Brick Township, New Jersey, Investigation.. Pediatrics.

[56] Rice CE, Baio J, Van Naarden BK, Doernberg N, Meaney FJ (2007). A public health collaboration for the surveillance of autism spectrum disorders.. Paediatr Perinat Epidemiol.

[57] Abrahams BS, Geschwind DH (2008). Advances in autism genetics: on the threshold of a new neurobiology.. Nat Rev Genet.

[58] O'Roak BJ, State MW (2008). Autism genetics: strategies, challenges, and opportunities.. Autism Res.

[59] Kusek GK, Wahlsten D, Herron BJ, Bolivar VJ, Flaherty L (2007). Localization of two new X-linked quantitative trait loci controlling corpus callosum size in the mouse.. Genes Brain Behav.

[60] Rosenberg RE, Law JK, Yenokyan G, McGready J, Kaufmann WE (2009). Characteristics and concordance of autism spectrum disorders among 277 twin pairs.. Arch Pediatr Adolesc Med.

[61] Dantzer R, Kelley KW (2007). Twenty years of research on cytokine-induced sickness behavior.. Brain Behav Immun.

[62] Stigler KA, Sweeten TL, Posey DJ, McDougle CJ (2009). Autism and immune factors: a comprehensive review.. Res Autism Spectr Disord.

[63] Ogura Y, Sutterwala FS, Flavell RA (2006). The inflammasome: first line of the immune response to cell stress.. Cell.

[64] Schmitz J, Owyang A, Oldham E, Song Y, Murphy E (2005). IL-33, an interleukin-1-like cytokine that signals via the IL-1 receptor-related protein ST2 and induces T helper type 2-associated cytokines.. Immunity.

[65] Sugama S, Conti B (2008). Interleukin-18 and stress.. Brain Res Rev.

[66] Wheeler RD, Brough D, Le Feuvre RA, Takeda K, Iwakura Y (2003). Interleukin-18 induces expression and release of cytokines from murine glial cells:interactions with interleukin-1β.. J Neurochem.

[67] Morale MC, Serra PA, L'episcopo F, Tirolo C, Caniglia S (2006). Estrogen, neuroinflammation and neuroprotection in Parkinson's disease: glia dictates resistance versus vulnerability to neurodegeneration.. Neuroscience.

[68] Brambilla P, Hardan A, di Nemi SU, Perez J, Soares JC (2003). Brain anatomy and development in autism: review of structural MRI studies.. Brain Res Bull.

[69] Rothwell NJ (1999). Annual review prize lecture cytokines-killers in the brain?. J Physiol.

[70] Bauer S, Kerr BJ, Patterson PH (2007). The neuropoietic cytokine family in development, plasticity, disease and injury.. Nat Rev Neurosci.

[71] Hudson CA, Christophi GP, Gruber RC, Wilmore JR, Lawrence DA (2008). Induction of IL-33 expression and activity in central nervous system glia.. J Leukoc Biol.

[72] Mondal TK, Saha SK, Miller VM, Seegal RF, Lawrence DA (2008). Autoantibody-mediated neuroinflammation: pathogenesis of neuropsychiatric systemic lupus erythematosus in the NZM88 murine model.. Brain Behav Immun.

[73] Heo Y, Costa LG, Hodgson E, Lawrence DA, Reed DJ (2005). In vitro model for modulation of helper T cell differentiation and activation.. Current Protocols in Toxicology.

[74] Milling SWF, Jenkins C, MacPherson G (2006). Collection of lymph-borne dendritic cells in the rat.. Nat Protoc.

[75] Fischer H-G, Reichmann G (2000). Brain dendritic cells and macrophages/microglia in central nervous system inflammation.. J Immunol.

[76] Campanella M, Sciorati C, Tarazzo G, Beltramo M (2002). Flow cytometry analysis of inflammatory cells in ischemic rat brain.. Stroke.

[77] Panchision DM, Chen HL, Pistollato F, Papini D, Ni HT (2007). Optimized flow cytometric analysis of central nervous system tissue reveals novel functional relationships among cells expressing CD133, CD15, and CD24.. Stem Cells.

[78] Paxonos G, Franklin KBJ (2004). “The Mouse Brain in Stereotaxic Coordinates. 2^nd^ Edition.

[79] Hafezi-Moghadam A, Thomas KL, Wagner DD (2007). ApoE deficiency leads to a progressive age-dependent blood-brain barrier leakage.. Am J Physiol Cell Physiol.

[80] Nadler JJ, Moy SS, Dold G, Trang D, Simmons N, Perez A (2004). Automated apparatus for quantitation of social approach behaviors in mice.. Genes Brain Behav.

[81] Gao D, Kasten-Jolly J, Lawrence DA (2006). The paradoxical effects of lead in interferon-gamma knockout BALB/c mice.. Toxicol Sci.

[82] Cao L, Lawrence DA (2002). Suppression of host resistance to Listeria monocytogenes by acute cold/resistant stress: lack of direct IL-6 involvement.. J Neuroimmunol.

